# On the Existence of a True Interparietal Bone in the Human Subject

**Published:** 1855-02

**Authors:** C. L. Hequembourg

**Affiliations:** Dansville, Liv. Co., N. Y.


### BUFFALO MEDICAL JOURNAL

AND

### MONTHLY REVIEW.

VOL. 10. FEBRUARY, 1855. NO. 9.

### ORIGINAL COMMUNICATIONS.

ART. I.— *On the Existence of a true Interparietal Bone in the Human Subject.* By Rev. C. L. Hequembourg, Dansville, Liv. Co., N. Y.

"Peruvian Antiquities. By M. E. Rivero, Director of the National Museum, Lima, etc.. and J. J. Von Tschudi, Doctor in Philosophy, Medicine, and Surgery, etc."

Dear Sir:—In the above-named work is a statement respecting the existence of a bone in the cranium of all the Peruvian races, and found in all their skulls; a figured specimen of which is given in the work. The bone is said to be placed between the two parietals, having a form more or less triangular, with the sharpest angle above, bounded by the posterior edges of the parietal bones, with its base attached to the occipital bone by a suture which runs from the angle of union of the temporal with the occipital bone, a little above the upper semicircular line, to the similar angle on the opposite side. In the figured specimen, which is said to be that of a youth of the Chinchas, ten or twelve years old, the length of the bone is four inches at the base and an inch and ten lines high.

The following observation is made upon this case by the authors: "It is a circumstance worthy of the attention of learned anthropologists, that there is thus found in one section of the human race a perpetual anomalous phenomenon, *which is wanting in all others,* but which is characteristic of the ruminant and carnivorous animals."—p. 39.

The American translator and editor adds the following note: "An anomaly characteristic of the ruminant and carnivorous animals would seem to exist in the ancient Peruvian crania. On this subject we are indebted to a professional friend, from whom we sought information, for the following note: '*The ossa Wormianæ* are small bones, found occasionally in all the sutures of the skull, but most frequently in the lambdoidal, which separates the occipital from the parietal bones. These *ossa Wormianæ* vary in size, from two lines to two inches in diameter. They are very variable in position and shape, as well as in size, and are mostly of an irregular form. The largeness of size, regularity of form, uniformity of shape, and position of the interparietal bones described by the author, distinguish them from the *ossa Wormianæ,* and seem to confirm his opinion that the ancient Peruvian skulls are peculiar, and mark a distinct and lower type of organization.'"—p. 41.

It will be observed that the American editor and his professional friend intimate their opinion that the author (rather authors, since the part upon the Peruvian crania, the Quichuan language, &c., was supervised and extended by Dr. Von Tschudi, at Vienna,) intended to restrict the peculiarity to the ancient Peruvians, from observations of their remains. That the authors did not quite intend this restriction is manifest from the pains taken to show that the original race is still in existence, with proof sufficient that their peculiarities cannot be altogether explained short of a reference to original constitution. The following extract will establish this opinion:

"We can therefore assert with certainty,

"I. That the race of the Chinchas is actually found, without any admixture, in various towns as well of the coast of Northern Peru, as of the province of the Yauyos.

"II. That the tribe of the Aymaræs is still found in the sierras of Southern Peru.

"III. That in some families of the department of Junin, the tribe of the Huancas is preserved pure, as we have had occasion ourselves to see.

"In conclusion it may be proper to notice an osteologic anomaly, very interesting, which is observed in the crania of all the three races."

The peculiarity in question is then particularly mentioned. See pp. 37, 38. We are aware that the professional note would be more favorably received by supposing the mummied remains are alone intended; but the work in the above continuous extract has spoken for itself.

An alleged fact, so remarkable, and with the extraordinary character assigned to it in this work, ought not to pass without observation; and the case falls both and indifferently within the province of the anatomist and of the theologian. Dr. J. C. Nott fully concurs with Rivero and Tschudi in the opinion that the peculiarities of the Peruvian skull are essentially natural. We will not say that he intends the bone in question, but in noticing the work with approbation, in the "Types of Mankind," (in the chapter upon "the Comparative Anatomy of Races,") he indicates his opinion that the cranial peculiarities of the Peruvians afford evidence that this Audian race are physically separated from the other families of mankind, and that they originated upon this continent out of the line of Adamic descent; in short, that they are the true "autocthones," indigenous to the continent. The theologian is interested in such a case, and the truth and dignity of anatomical science and of anthropology not less. As the case of the theologian, however, is one of graver interest, it is not improper that it should specially attract his attention, if in no other way than in that of calling the attention of anatomists to the subject for its more complete elucidation. It may not offend your medical readers to receive some observations upon this case from a profession which cannot be supposed to be well furnished with anatomical knowledge; the two professions meet in the same cases of suffering, and may sometimes meet, and often do, as amicably, in the pages of a Journal.

It is a remarkable and not very prepossessing feature in this case, that the peculiarity to which such grave consequences are attached is not usually mentioned in the common and reliable works upon human anatomy. The same is true of the great works upon ethnology. It is not noticed in Prichard's Natural History of Man; nor in the great German compilation of Heck, founded upon the Bilder Atlas.* The foundation of Dr. Prichard's view of the Peruvian branch of the Audian family seems to be derived in great part from the investigations of M. d'Orbigny; but neither does this great naturalist and ethnologist mention the peculiarity so far as reference is made to his researches by Prichard. The subject, therefore, does not present itself with the entire reliableness which would satisfy the most scrupulous readers. Even the alleged fact itself, aside from the deductions, might be liable to very considerable modifications, if it were found to be true at all as a universal peculiarity. The authors do not take the care to inform us whether the bone is always of the size of the figured specimen; nor does Dr. Von Tschudi inform

* "Iconographic Encyclopædia, in 4 vols., with 500 plates, and upward of 12,000 engravings."

us upon a point upon which he must have been capable of deciding, whether the peculiarity belongs to both tables of the cranium, nor whether the bone is always found single and never composed of several pieces: questions, certainly some of them, of great importance. And in regard to the uniform existence of the bone in question, we may reasonably doubt the ability of any man to decide absolutely upon such a case, since we cannot suppose that the materials of the judgment are completely accessible; notwithstanding the remarkable adaptedness of the climate of Peru to preserve the remains of man, the corpses of kings were better preserved than those of others, and this fact may lead to the suspicion that a meaner class of people, whose cranial contour was not conformed by artificial means to the standard of courtly taste, might have been as free from the peculiarity in question (viewed as the result of pressure in infancy) as some of the learned inhabitants of Peru are at the present day. We will not, however, discuss this point. Not possessing the means of testing the entire accuracy of the statement, we proceed in noticing the case, upon more general grounds. The force of the reasoning, even admitting the fact to be altogether true, falls more within the scope of our power.

Admitting, then, the existence of an interparietal bone, as described, and so called, — the name is nothing, — how are the deductions supported from this fact, viz., that the original Peruvian races are marked by a low type of organization, and chiefly because they possess a characteristic of the ruminant and carnivorous animals, and that their cranial peculiarity is not to be regarded as belonging to the class of the *ossa Wormianæ?*

I. There are known to be numerous departures from uniformity, within a limited extent of variation, in almost every part, it might be said, of the human body; and some of these might be regarded as suggestive of something, for the want of a better comparison, in the inferior animal creation. But does it necessarily follow that a man is specifically or essentially lower in organization, because his toes are webbed, or his skin has the external appearance of the legs of a rhinoceros as in the case of Machin and family? It would be mere fancy to denominate accidents of this description as *organization.* The variations from normal forms constantly produced by disease, might with as real propriety, all be referred to human organization. It can make little difference that some variations should be extended over a whole tribe, of whose historical origin we knew nothing, as is certainly the case in some remarkable cranial configurations, instead of being confined to one person or family; since very striking differences may become persistent, as peculiarities of features have been transmitted for several generations notwithstanding repeated crossing: otherwise the features of the *os calcis* in some persons, and in extensive varieties of the human race, should be regarded as in itself sufficient evidence of a specifically lower organization—for we may presume that specific difference or difference enough to establish a plurality of origin to the human race is what the professional note intends; but this peculiarity of a flat foot is found diffused through the European, and perhaps all other races, so that the specific character of the peculiarity is lost. So it might be said that the greater proportional length of the forearm in some persons approximated them to the ape, and that the relative posterior position of the foramen magnum in some Europeans and Americans, as measured from the front of the upper jaw, was evidence of inferior organization, and rendered the ape or the chimpanze the type of some men. Upon the same principles of reasoning the occasional appearance of cartilages in the different tissues of the body, and the occasional existence of bony plates, might, for aught we see, be regarded as denoting the degradation of the human organization and the return of man to the state of a fish, with cartilages preparing to be bone, and bones without support. We shall not dispute with the comparative anatomist any indication which some singularity of the human body may be supposed to make of some great archetypical plan of the Creator, to be developed in another state; but accidental cartilages and bony scales do not certainly denote either that man was once a fish nor probably that he is tending to become one, and is likely hereafter to swim in the watery realms of Jupiter or Saturn.*

II. The existence of some variety in individual man, or in races, which may have something resembling it in the lower creation, as in birds, for instance, or the pachydermata or the ourang, does not then necessarily denote in any proper sense a lower organization.† This principle might be applied on a more extensive scale to the great questions of ethnology; but we shall

---

* See Plurality of Worlds.

† We take the liberty of interpolating here an extract from "Owen on the Skeleton and Teeth," which, while it recognizes the analogies of different races, harmonizes them with the principles of Christianity by showing in these resemblances, not a law of progression in development, but a chain of likenesses, resulting from the fact that one Creator formed all living beings on one plan, or archetype, upon the modifications of which all individuality, as well as all difference, depends.—Ed. Buff. Med. Jour.

"A retrospect of the varied forms and proportions of the skeletons of animals, confine ourselves to the case in hand, and inquire whether the Peruvians possess such an organization as to make it proper to compare them with the ruminant or carnivorous animals for the sake of showing by this analogy or affinity, or whatever may be meant, an inferior nature. We need not em-

---

whether modified for aquatic, aerial, or terrestrial life, will show that whilst they were perfectly and beautifully adapted to the sphere of life and exigences of the species, they adhered with remarkable constancy to that general pattern or archetype which was first manifested on this planet, as Geology teaches, in the class of fishes, and which has not been departed from even in the most extremely modified skeleton of the last and highest form which Creative Wisdom has been pleased to place upon this earth.

"It is no mere transcendental dream, but true knowledge and legitimate fruit of inductive research, that clear insight into the essential nature of each element of the bony framework, which is acquired by tracing them step by step, as *e. g.* from the unbranched pectoral ray of the lepidosiren to the equally small and slender but bifid pectoral ray of the amphiume, thence to the similar but trifid ray of the proteus, and through the progressively superadded structures and perfections of the limbs in higher reptiles and in mammals. If the special homology of each part of the diverging appendage and its supporting arch are recognizable from man to the fish, we cannot close the mind's eye to the evidences of that higher law of archetypal conformity on which the very power of tracing the lower and more special correspondences depend.

"Buffon has well remarked, in the Introduction to his great work on Natural History: 'It is only by comparing that we can judge, and our knowledge turns entirely on the relations that things bear to those which resemble them and to those which differ from them; so, if there were no animals, the nature of man would be far more incomprehensible than it is.'

"And if this be true, as to man's general nature and powers, it is equally so with regard to his anatomical structure.

"In the same spirit our philosophic poet felt that —

> ''T is the sublime of man
> Our noontide majesty, to know ourselves
> Part and proportions of a wondrous whole.'—Coleridge.

"Vertebrated animals of progressively higher grades of structure have existed at successive periods of time on this planet, and they were constructed on a common plan with those that still exist.

"Some have concluded, therefore, that the characters of a species became modified in successive generations, and that it was transmuted into a higher species; a reptile, *e. g.* into a mammal; an ape, into a negro. Let us consider, therefore, the import and value of the osteological differences between the gorilla — the highest of all apes — and man, in reference to this 'transmutation hypothesis.' The skeleton of an animal may be modified to a certain extent by the action of the muscles. By the development of the processes, ridges, and crests, the anatomist judges of the barrass ourselves with the task of spelling out the meaning of the authors or of the professional friend, we may take the case in the broad light in which a careless juxtaposition of ideas or intention may permit, perhaps require,

---

muscular power of the individual to whom a skeleton under comparison has appertained. A very striking difference from the form of the human cranium results from the development of certain crests and ridges for the attachment of muscles, in the great apes; but none of the more important differences, on which the naturalist relies for the determination of the genus and species of the orangs and chimpanzees, have such an origin or dependent relation. The great superorbital ridge, *e. g.* against which the facial line rests, in Fig. 50, is not the consequence of muscular action or development: it is characteristic of the genus *Troglodytes* from the time of birth; and we have no grounds for believing it to be a character to be gained or lost through the operations of external causes, inducing particular habits through successive generations of a species.

" No known cause of change productive of varieties of mammalian species could operate in altering the size, shape, or connections of the prominent premaxillary bones, which so remarkably distinguish the great *Troglodytes gorilla* from the lowest races of mankind. There is not, in fact, any other character than that founded upon the development of bone for the attachment of muscles, which is known to be subject to change through the operation and influence of external causes. Nine-tenths of the differences which have been cited (see the *Transactions of the Zoological Society*, vol. iii. p. 413,) as distinguishing the great chimpanzee from the human species, must stand in contravention of the hypothesis of transmutation and progressive development, until the acceptors of that hypothesis are enabled to adduce the facts demonstrative of the conditions of the modifiability of such characters. Moreover, as the generic forms of the ape tribe approach the human type, they are represented by fewer species. The unity of the human species is demonstrated by the constancy of those osteological and dental peculiarities which are seen to be most characteristic of the *bimana* in contradistinction from the *quadrumana*.

" Man is the sole species of his genus (*homo*) — the sole representative of this order (*bimanæ*); he has no nearer physical relations with the brute kind than those that belong to the characters which link together the unguiculate division of the mammalian class.

" Of the nature of the creative acts by which the successive races of animals were called into being, we are ignorant. But this we know, that as the evidence of unity of plan testifies to the oneness of the Creator, so the modifications of the plan for different modes of existence illustrate the beneficence of the Designer. Those structures, moreover, which are at present incomprehensible as adaptations to a special end, are made comprehensible on a higher principle, and a final purpose is gained in relation to human intelligence; for in the instances where the analogy of humanly invented machines fails to explain the structure of a divinely created organ, such organ does not exist in vain if its truer comprehension, in relation to the Divine idea, or prime Exemplar, leads rational beings to a better conception of their own origin and Creator."—Vide page 225 of Owen on Skeleton and Teeth.

us. Cuvier, then, maintained, and established, that the examination of the bones of the head or of the feet, or indeed of any bone in the animal skeleton, was sufficient to decide in regard to the character of the animal to which it belonged, because the principal characteristic of an animal impresses itself upon every bone in the body. Now the application of this law to the present case, shows the gross anatomical error of the statements. If the Chinchas or Aymaraes of Peru, possess a characteristic which allies them in any manner properly to the ruminant or carnivorous brutes, *indicative of a lower organization,*—for this is the statement and the reason,—it ought not to be unattended with other peculiarities, otherwise the comparison is trifling with language and with ideas. It would be absurd to pursue the inquiry whether the Peruvians, so highly characterized for civilization, for the art of sculpture, the computation of the solar year, and systems of law, were physically inferior to the Ojibwas and other American Indians, or whether they possessed any ruminant faculty or carnivorous prehensiles. It is unfortunate that the case should have been one characteristic of so much civilization. We need only imagine—if these ideas should be regarded as rather sportive—what would have been the judgment of Cuvier in witnessing the head figured in the work of Rivero and Tschudi, and believe that he would have immediately set it down as a variety not at all affecting the question of specific diversity or even of degradation of organization. One of the medical gentlemen of the town where the author resides, Dr. Endress, recollects seeing the skull of a white person which had the very peculiarity mentioned by these writers, in the existence of a large bone, about two inches in extent, occupying the place of the upper portion of the occipital. We further notice only the statements of Prichard in summing up the peculiarities of the Inca-Peruvians—living specimens of whom, or of the root from which they sprung, being in existence, the authors tell us, now. "The forehead is narrow, but the vertex elevated: these traits, and the shortness of the antero-posterior diameter" (something very different from the skulls of ruminant or carnivorous animals) "are the principal characters of the skull of the ancient Incas." Dr. Prichard was well acquainted with the labors of Morton in assigning the peculiarities of the Peruvian and American crania.

III. The question only remains whether the interparietal bone, so named, is an *os Wormianum.* This question scarcely belongs to the present writer to touch, and may be better left to the eminent professors of your University. Besides, the question is perhaps not essential. It is admitted by every one, even by the authors of the book in hand, that the Peruvians used a custom, so common to the American races, of altering the natural shape of the head by artificial means. Indeed this is a historical fact, since the practice was prohibited among the Peruvians by a bull of the Pope. How far such a practice would affect the formation of bone may be left to anatomists and physiologists to decide. Heads with an irregular contour, for which the American races are remarkable, are, as is well known, most liable to the formation of unusual bones. Some Indian skulls possess very large and numerous *ossa Wormianæ* in the lambdoidal suture, sometimes very glaringly represented in engraved specimens. In the pressure of the head, practiced by the Peruvians, M. d'Orbigny observes that the brain was forced backward by a force applied in some manner to the frontal bones; a process by which the posterior portions of the parietals and the occiput would be much affected. This process performed in infancy, might naturally change the formations of the occiput as they arose from their different centres of ossification, from the increase of the transverse diameter. At any rate a greater space than usual behind the parietals would be required, from the backward and upward pressure, to be filled up; and in this case a greater *os Wormianum* would exist, and that would be all. Whether any parts of the occipital bone might be united by sutures and partly or wholly suppressed by muscular pressure is a question of anatomy which the writer would not presume to decide: perhaps such a position would stand opposed to the opinion, perhaps commonly received, of existence of original septa in the lamina between which the bony plates of the skull are formed. We may nevertheless ask with what propriety the bone in question can be called interparietal. It does not lie between the parietals alone, but is posteriorly connected to them. The name, therefore, as suggesting the idea of lying between, is improper and false; and we leave it to comparative anatomists to decide whether the comparison implied and intended is any less so, in other words whether a bone taking the place of the upper portion of the occipital, corresponds with the interparietal bone of the lower animals.

After this article was sketched and nearly completed, a copy of Cruveilhier's Anatomy was met with, who represents the so-called interparietal bone as a true complimentary bone, and speaks of it without reference to Peruvian crania, leaving it to be inferred that the case is not peculiar to this race. He also observes that "in some skulls the whole of the occipital bone above the occipital protuberance is formed by these (complimentary) bones."

